# Quality of care in dysphagia patients: adaptation and validation of the Swedish SWAL-CARE questionnaire

**DOI:** 10.1186/s12955-020-01562-2

**Published:** 2020-09-25

**Authors:** Johanna Hedström, Mia Johansson, Caroline Olsson, Lisa Tuomi, Caterina Finizia

**Affiliations:** 1 Department of Otorhinolaryngology, Head and Neck Surgery, Institute of Clinical Sciences, Sahlgrenska Academy, Gothenburg University, 413 45 Gothenburg, Sweden; 2 Department of Anesthesia and Intensive Care, Region Västra Götaland, Sahlgrenska University Hospital, Area 2, 416 85 Gothenburg, Sweden; 3 Department of Oncology, Institute of Clinical Sciences, Sahlgrenska Academy, Gothenburg University, 413 45 Gothenburg, Sweden; 4 Department of Oncology, Region Västra Götaland, Sahlgrenska University Hospital, 413 45 Gothenburg, Sweden; 5 Department of Radiation Physics, Institute of Clinical Sciences, The Sahlgrenska Academy Gothenburg University, 413 45 Gothenburg, Sweden; 6 Regional Cancer Center West, The Western Sweden Healthcare Region, Sahlgrenska University Hospital, 413 45 Gothenburg, Sweden; 7 Department of Otorhinolaryngology, Region Västra Götaland, Sahlgrenska University Hospital, 413 45 Gothenburg, Sweden

**Keywords:** Deglutition disorders, Quality of care, Questionnaires, Validation studies, Psychometrics, SWAL-CARE

## Abstract

**Background:**

The aim of this study was to adapt the instrument and evaluate the psychometric properties of the Swedish version of the Swallowing Quality of Care questionnaire (S-SWAL-CARE) in patients with oropharyngeal dysphagia.

**Methods:**

Translation and adaptation of the original SWAL-CARE into Swedish was performed according to established international guidelines. Field testing was performed using 100 patients with oropharyngeal dysphagia due to multiple reasons such as head and neck cancer and neurologic/neuromuscular disease, who had undergone swallowing evaluation within 6 months prior to the study. The patients answered the S-SWAL-CARE, the Quality from the Patient’s Perspective (QPP) and the Swallowing Quality of Life (SWAL-QOL). Test–retest was performed in 20% of the participants. The reliability and validity of the S-SWAL-CARE were assessed by Pearson correlation coefficient and Cronbach’s alpha as well as convergent and discriminative validity, respectively.

**Results:**

The field testing of the S-SWAL-CARE resulted in sufficient reliability, with Cronbach’s alpha values exceeding 0.90 for all domains. All items correlated strongly to their own domain, with weaker correlations to the other domains, indicating proper scale structure. Results also indicate sufficient convergent and discriminant validity when tested for association to the QPP domains and the SWAL-QOL Total score. The test–retest reliability of the S-SWAL-CARE demonstrated sufficient intraclass correlation coefficient (ICC) for the General advice domain (0.73) and Clinical advice domain (0.82). The ICC for the Patient satisfaction domain was lower (0.44).

**Conclusion:**

The S-SWAL-CARE can be considered a reliable and valid tool to assess the dysphagia-related quality of care in a mixed Swedish dysphagia patient population.

## Background

Swallowing impairment (dysphagia) involves difficulties transferring saliva, food and liquids from the oral cavity to the stomach and is a common and troublesome condition in the general population, primarily among individuals over 60 years old (circa 40%) [1]. Dysphagia may afflict patients with neurological disease, vascular disease (i.e. stroke), traumatic head injury or head and neck cancer (HNC), and the patients’ general health as well as quality of life is affected [2]. Common and serious complications to dysphagia are malnutrition and dehydration as well as aspiration with possible pneumonia as a consequence [3]. It is therefore of great importance to ensure early diagnosis and offer adequate treatment i.e. individually-adapted compensatory interventions to ensure safe swallowing.

Besides clinical diagnostic evaluation of the swallowing function, it is also important to evaluate the patient’s perception of swallowing and experience of the given treatment and care. This can be done by using Patient Reported Outcome Measures (PROM) instruments, i.e. validated questionnaires evaluating the patient’s own perception of their health status. In the United States, diagnosis-specific instruments have been developed for the dysphagia population. The Swallowing Quality of Life questionnaire (SWAL-QOL) [4–7] and the M. D. Anderson Dysphagia Inventory (MDADI) [8] are two examples of instruments used in clinical research and practice to describe the degree of dysphagia and evaluate the effect of dysphagia treatment. In addition to the quality of life (QOL) aspect, it has become important to evaluate the quality of care from the patient’s perspective.

Quality of care is a concept measuring what aspects of the care that the patients consider important as well as their satisfaction with the care given [9]. It brings the opportunity for care givers to get feedback on the given treatment and care. If patients are not content and do not have confidence in the provided care, there is a great chance that they will not be compliant with the given advice and the risk for dysphagia complications could increase. McHorney et al. have developed a Patient Reported Experience Measure (PREM) instrument for dysphagia, the Swallowing Quality of Care questionnaire (SWAL-CARE). To our knowledge there is no other instrument measuring quality of care in dysphagia.

The SWAL-CARE evaluates the patient’s opinion on received clinical information and swallowing safety advice as well as patient satisfaction, and it can be used to improve the care for dysphagia patients. This instrument was originally a part of the SWAL-QOL, but in the final development phase the multi-item instrument was separated into two patient-centered outcome tools [7]. The original SWAL-QOL [7] has been translated and validated in several languages [10–15], but the original SWAL-CARE has to this date not been adapted into another language.

To the best of our knowledge, no instrument evaluating quality of care in dysphagia patients exists in Swedish. The aim of this study was therefore to translate the SWAL-CARE into Swedish (S-SWAL-CARE) and evaluate the psychometric properties of this adapted version in patients with oropharyngeal dysphagia.

## Methods

### Patient cohort and study protocol

Patients eligible for inclusion in the field testing of the S-SWAL-CARE were identified through the outpatient booking system at the otorhinolaryngology clinic at the Sahlgrenska University Hospital in Gothenburg. Patients ≥ 18 years old with oropharyngeal dysphagia who had undergone swallowing evaluation at the outpatient clinic through videofluoroscopy, fiberoptic endoscopic evaluation of swallowing (FEES) or clinical evaluation of swallowing by a speech and language pathologist (SLP) within the last six (6) months, were identified. Patients were excluded if they were not able to answer the questionnaires due to insufficient knowledge in the Swedish language or if they had severe cognitive impairment. The desired sample size was 100 participants in accordance to guidelines by Fayers and Machin [16] where five to ten respondents per item are required.

The eligible study participants were contacted by telephone and included in the study if they gave their written informed consent to participate. The included patients were then sent the study questionnaires by mail or e-mail. The study participants were reminded once by telephone if they had not returned the filled-out questionnaires within 2 weeks.

### Study instruments

In this validation study, three patient-reported instruments were used; two on quality of care, namely, the S-SWAL-CARE and the Quality from the Patient’s Perspective (QPP) [17, 18]; and one on quality of life related to oropharyngeal dysphagia, the S-SWAL-QOL. Supplementary questions regarding difficulties swallowing, eating, drinking and coughing when eating/drinking as well as questions on patient demographics were also used.

#### The SWAL-CARE

The SWAL-CARE is a 15-item tool which assesses quality of care and patient satisfaction among patients with oropharyngeal dysphagia. The items are divided into 3 domains: Clinical advice (items 1–6), General advice (items 7–11) and Patient satisfaction (items 12–15). Items 1–11 are answered on a scale from 1–6, where 1 = bad; 6 = excellent, and items 12–15 are answered on a scale from 1–4, where 1 = never; 4 = always. Domain scores are calculated by linear transformation to a range from 0 to 100, with 0 indicating the least favorable and 100 the most favorable quality of care. Scores are calculated according to the directions of the original SWAL-CARE, where domain scores are calculated if more than 50% of the items of the domain are answered. All domains in the original SWAL-CARE have been shown to exhibit excellent internal consistency reliability and sufficient short-term reproducibility [7].

The original SWAL-CARE questionnaire was translated into Swedish using a formal forward–backward translation method, according to international translation guidelines [16, 19, 20]. Two Swedish native speakers with advanced knowledge and understanding of English, informed in medical care and the terminology of the questionnaire, performed independent translation of the English version into Swedish. Next, a bilingual expert panel with great knowledge in the field as well as translation and adaptation of questionnaires, combined the two versions into a consensus version. The wording of parts of the questionnaire instructions was slightly modified to allow better coherence with the existing dysphagia care in Sweden. The consensus version was then retranslated to the original language by an independent bilingual individual with English as native language and unfamiliar with the questionnaire. The backward translation of the S-SWAL-CARE did not reveal any differences from the original SWAL-CARE.

A pilot study including 10 adult dysphagia patients was performed, selected by the same criteria as the field testing cohort. The study participants were sent the study questionnaires and 1 week after the answered questionnaires were received, a semi-structured telephone interview was conducted. The interview was a complementary review of how the S-SWAL-CARE was perceived by the respondents. Based on the results of the interviews the S-SWAL-CARE was revised and a final version was established. In the pilot study, several patients declared the need for an additional response alternative indicating that the question did not apply to them, for example if they did not receive advice from the swallowing clinician. Therefore, an additional response alternative “Did not receive any advice” was added to the instrument. This response alternative was not included in the calculation of item and domain scores.

#### The quality from the patient’s perspective (QPP)

The QPP is an instrument on quality of care developed and validated in Sweden and it has been adapted to several different health care establishments [17, 18]. In collaboration with the owners of the questionnaire (IMPROVEIT), the QPP was adapted to be compatible with the care given by swallowing specialists at the otorhinolaryngology clinic at the Sahlgrenska University Hospital. The QPP consists of 19 items which are answered from two perspectives: perceived reality of quality of care (I have had…) and subjective importance (This is how important it was for me to have…). In the validation of the S-SWAL-CARE questionnaire only the “perceived reality” perspective will be used since this perspective matches how the items in the S-SWAL-CARE are phrased. Items 1–16 form four domains: Medical-technical competence, Physical-technical conditions, Identity-oriented approach and Socio-cultural atmosphere. The items are answered on a scale from 1–4, where 1 = Completely disagree; 4 = Completely agree. A mean score of the items in each domain is calculated, forming the domain score ranging from 1–4 where one (1) indicates poor quality of care and four (4) indicates very good quality of care. In the validation study of the S-SWAL-CARE, the QPP was used to compare the S-SWAL-CARE to a questionnaire measuring a similar phenomenon, i.e. the convergent validity.

#### The SWAL-QOL

The SWAL-QOL [7] is considered the gold standard for evaluating quality of life in individuals with oropharyngeal dysphagia [10, 11]. It has been translated into Swedish and validated by Finizia et al. [11]. The SWAL-QOL is a 44-item instrument divided into 10 domains: General burden, Eating desire, Eating duration, Food selection, Communication, Fear, Mental health, Social functioning, Fatigue, Sleep. There is also a symptom-frequency domain, Symptom scale. The SWAL-QOL Total score is calculated from general burden, food selection, mental health, social functioning, fear, eating duration and eating desire domains. Domain scores are calculated by linear transformation to a range from zero to 100, indicating an extremely impaired quality of life (0) versus no impairment (100), as experienced by the individual. The domains differentiate normal swallowers from patients with oropharyngeal dysphagia and are sensitive to differences in the severity of dysphagia [7]. The SWAL-QOL has been shown to exhibit good internal-consistency reliability and short-term reproducability [7].

In the validation of the S-SWAL-CARE, the SWAL-QOL was primarily used as a measure of dysphagia severity and does not reflect the clinical care given, as in the S-SWAL-CARE.

#### Study-specific questions

The study participants also answered questions to evaluate the feasibility of the S-SWAL-CARE, i.e. time used to fill out the questionnaire, if they needed help to fill out the questionnaire and if any question made them anxious/disturbed. They also answered the following questions: “Do you have problems eating, drinking or swallowing? Do you cough when eating/drinking?

### Statistical analyses and psychometric testing

Descriptive statistics were used to summarise the demographical and clinical characteristics of the study participants. The distribution of the variables was given as mean, standard deviation (SD), median and range for continuous variables and as numbers and percentages for categorical variables. The added response alternative “Did not receive any advice” was treated as missing data since it was not included in the calculations of item and domain scores.

All statistical analyses were performed using the statistical software SAS^®^ System version 9.4 (SAS Institute Inc., Cary, NC, USA) (if not stated otherwise). All tests were two-tailed and conducted at a 5% significance level.

#### Reliability and reproducibility

The correlation between each item and its respective domain was assessed through Pearson’s correlation coefficient (Pr): Pr ≤ 0.39 correspond to weak correlation; 0.4–0.59 moderate correlation; ≥ 0.6 strong correlation [21]. Internal consistency reliability was calculated by means of Cronbach’s alpha coefficient (range 0–1). Cronbach’s alpha estimates > 0.7 were taken to indicate sufficient internal consistency reliability [16]. Test–retest reliability, as assessed by intraclass correlation (ICC), was evaluated for 20 study participants by repeated comparison of the S-SWAL-CARE at the time of enrollment and 2 weeks after. The 2-week-long interval period was selected since no substantial change in swallowing function was expected to occur within this period. No access to the responses given in the first questionnaire was granted to patients filling out the second S-SWAL-CARE. An ICC value of 0.4–0.75 corresponds to moderate to good reliability and a value > 0.75 corresponds to very good reliability [22, 23]. Reliability estimates greater than 0.7 were considered to be the minimal acceptable/satisfactory correlation for group-level comparison [24].

#### Validity

Construct validity include if the instrument measures the intended constructs and consists of convergent and discriminant validity [16]. The Spearman correlation coefficient (ρ) was used to assess convergent and discriminant validity: < 0.3 was considered to be a weak correlation, 0.3–0.7 moderate correlation, and > 0.7 a strong correlation [25]. Convergent validity is assessed by comparing the respective items and domains in the S-SWAL-CARE with the items and domains in the QPP. Discriminant validity was evaluated through comparison of the S-SWAL-CARE domains to the SWAL-QOL Total score. We hypothesized a priori that the Clinical advice domain would show moderate correlations to the QPP domains, with the highest correlations for the Medical-Technical competence domain. For the General advice domain, we hypothesized moderate correlations to all the QPP domains. Of the three S-SWAL-CARE domains, the Patient satisfaction domain was hypothesized to show the strongest correlations to the QPP domains, with the strongest correlations to the Identity-oriented approach and Socio-cultural atmosphere domains. All S-SWAL-CARE domains were expected to show weak correlations to the SWAL-QOL Total score. For explicitness, in the Results and Discussion sections correlations are reported as “r”, regardless of them being Pearson’s or Spearman’s correlation coefficients.

Floor and ceiling effects measure the proportion of patients having the minimum or maximum score, respectively. The presence of floor and ceiling effects, in which > 15% (as suggested by McHorney et al. [26]) of the patients grade themselves as having the maximum or minimum score, indicates that the response scale will have poor discrimination and that the item might need to be reconstructed.

### Ethics, consent and permissions

The study was conducted in accordance with the Declaration of Helsinki and was approved by the Regional Ethical Review Board in Gothenburg, Sweden. All participants gave their written informed consent before inclusion in the study.

## Results

### Patient characteristics

Two hundred and sixty-one patients were eligible for inclusion in the study, 46 patients were excluded (inpatient examination n = 33; insufficient knowledge in Swedish n = 12; severe cognitive impairment n = 1) leaving 215 patients eligible for inclusion in the study. Upon dial-up, 49 patients could not be contacted and 66 patients declined participation in the study (difficulty filling out questionnaire/too many questions n = 4, did not think that they were part of the target group n = 6, did not think that they received any help n = 1, reduced general condition n = 13, unspecified n = 42). One hundred patients were included in the validation study. Table 1 lists the socio-demographic and clinical characteristics of the study participants. Of the 100 study participants, 56% were men and 44% women with a median age of 70 years (range: 23–95). Primary causes of dysphagia were neurologic or neuromuscular disease (43%), head and neck cancer (28%) and vascular disease i.e. stroke (3%). Other causes were for example mainly esophageal symptoms, psychogenic dysphagia, chronic obstructive pulmonary disease or unclear cause of dysphagia. Regarding the swallowing examination carried out, 81/100 (81%) of the study participants went through fiberoptic endoscopic evaluation of swallowing (FEES). Seventy-four percent of the study participants experienced swallowing difficulties (Table 1).

**Table 1:** Socio-demographic and clinical characteristics of the study participants

Variable	N = 100
Age, years (median/range)	70 (23–95)
	n (%)
Age categories	
18–55 years	19 (19)
> 55–70 years	33 (33)
> 70–85 years	43 (43)
> 85 years	5 (5)
Gender	
Male	56 (56)
Female	44 (44)
Marital status	
Single	28 (28)
Married/cohabitant	70 (70)
Partnership but living apart	2 (2)
Education level	
Elementary school	31 (31)
High school	24 (24)
University up to 4 years	24 (24)
University > 4 years	21 (21)
Working status	
Retired	62 (62)
Unemployed	6 (6)
Yes, fulltime	14 (14)
Yes, part time	18 (18)
Smoking status	
Never smoked	38 (38)
Previous smoker	54 (54)
Current smoker	8 (8)
Primary cause of dysphagia	
Cancer	28 (28)
Vascular disease (i.e. stroke)	3 (3)
Neurologic or neuromuscular disease	43 (43)
Other cause	26 (26)
Swallowing examination carried out	
Fiberscopic evaluation of swallowing	81 (81)
Videofluoroscopy	9 (9)
Clinical swallowing examination	10 (10)
Eating difficulties	55 (55)
Drinking difficulties	44 (44)
Swallowing difficulties	74 (74)
Coughing when eating/drinking	62 (62)
Body Mass Index (BMI) kg/m^2^ (median/range)	24.2 (16.7–35.8)

### Feasibility

The study participants completed the S-SWAL-CARE questionnaire at home and returned them by mail (n = 90; 90%) or by e-mail via an electronic version of the questionnaire (n = 10; 10%). It took 10–20 min for most of the study participants to complete the questionnaire. Twenty of the respondents (20%) needed assistance to complete the questionnaire. The majority was assisted by someone else reading the questions and/or writing down the answers. In total, 36 patients reported that a question was difficult to understand, but most of them did not specify why. There was not one specific question reported as difficult to understand, i.e. the items specified as difficult to understand was evenly distributed over the three domains. In the cases that they did state why it was difficult to respond to the specified question, it was mainly due to that they had seen a swallowing specialist several times, and it was difficult to know which occasion they should refer to. Mainly, this concerned items of the Patient satisfaction domain. When stating that a question made them upset or disturbed (n = 6), they did not specify which question they referred to.

### Reliability and reproducibility

The SWAL-CARE domains and items with abbreviations are listed in Table 2 in order to simplify the understanding of the presented results in the tables. All of the S-SWAL-CARE domains showed good internal consistency reliability with a Cronbach’s alpha ≥ 0.90. All items were strongly correlated with its respective domain (r ≥ 0.81 for all) (Table 3).

**Table 2:** The SWAL-CARE domains and items with abbreviations

Domain	Item	Abbreviation used in tables
Clinical advice	1. Foods I should eat	Foodeat
	2. Foods I should avoid	Foodavoid
	3. Liquids I should drink	Liqdrink
	4. Liquids I should avoid	Liqavoid
	5. Techniques to help me get food down	Fooddown
	6. Techniques to help me avoid choking	Avoidchk
General advice	7. When I should contact a swallowing clinician	Contact
	8. Goals of the treatment for my swallowing problem	Goals
	9. My treatment options	Options
	10. What to do if I start to choke	Dochoke
	11. Signs that I am not getting enough to eat or drink	Signs
Patient satisfaction	12. You had confidence in your swallowing clinicians	Confid
	13. Your swallowing clinicians explained everything about your treatment to you	Explain
	14. Your swallowing clinicians spent enough time with you	Clintime
	15. Your swallowing clinicians put your needs first	Myneeds

**Table 3:** Reliability estimates for the S-SWAL-CARE

S-SWAL-CARE domain	Item label	Correlation with its domain	Cronbach’s alpha
Clinical advice domain	1. Foodeat	0.85	0.92
	2. Foodavoid	0.87	
	3. Liqdrink	0.85	
	4. Liqavoid	0.86	
	5. Fooddown	0.83	
	6. Avoidchk	0.86	
General advice domain	7. Contact	0.88	0.90
	8. Goals	0.87	
	9. Options	0.81	
	10. Dochoke	0.84	
	11. Signs	0.85	
Patient satisfaction domain	12. Confid	0.91	0.94
	13. Explain	0.92	
	14. Clintime	0.95	
	15. Myneeds	0.93	

For the Clinical advice domain, the items demonstrated strong Pearson correlations to their own domain ranging from 0.79–0.86 (Table 4). The Pearson correlations of the items to the other two domains were consistently lower, ranging from 0.33–0.74. The same was demonstrated for the items of the General advice domain and Patient satisfaction domain. Scaling success was defined as when an item correlated significantly higher to their own domain than the other domains. For the General advice domain and the Patient satisfaction domain 100% of all items in the own domain were significantly more correlated to its own domain than to the other domains. For the Clinical advice domain, the scaling success rate was 83%.

**Table 4:** Pearson correlation between SWAL-CARE items and domains

SWAL-CARE item/domain	Clinical advice domain	General advice domain	Patient satisfaction domain
	r (95% CI)	r (95% CI)	r (95% CI)
1. Food eat	*r* *=* *0.79 (0.68–0.86)*	r = 0.67 (0.50–0.79)	r = 0.33 (0.12–0.51)
	*p* *=* *<* *0.0001*	*p* = < 0.0001	*p* = 0.0024
2. Food avoid	*r* *=* *0.81 (0.72–0.88)*	r = 0.66 (0.48–0.79)	r = 0.37 (0.15–0.55)
	*p* *=* *<* *0.0001*	*p* = < 0.0001	*p* = 0.0014
3. Liqdrink	*r* *=* *0.82 (0.73–0.88)*	r = 0.74 (0.60–0.84)	r = 0.38 (0.17–0.56)
	*p* *=* *<* *0.0001*	*p* = < 0.0001	*p* = 0.0007
4. Liqavoid	*r* *=* *0.86 (0.78–0.91)*	r = 0.74 (0.58–0.84)	r = 0.39 (0.16–0.58)
	*p* *=* *<* *0.0001*	*p* = < 0.0001	*p* = 0.0012
5. Fooddown	*r* *=* *0.83 (0.75–0.89)*	r = 0.73 (0.59–0.83)	r = 0.39 (0.18–0.57)
	*p* *=* *<* *0.0001*	*p* = < 0.0001	*p* = 0.0004
6. Avoidchk	*r* *=* *0.85 (0.77–0.90)*	r = 0.72 (0.56–0.82)	r = 0.38 (0.17–0.56)
	*p* *=* *<* *0.0001*	*p* = < 0.0001	*p* = 0.0006
7. Contact	r = 0.72 (0.56–0.83)	*r* *=* *0.88 (0.80–0.93)*	r = 0.49 (0.25–0.67)
	*p* = < 0.0001	*p* *=* *<* *0.0001*	*p* = 0.0001
8. Goals	r = 0.74 (0.61–0.83)	*r* *=* *0.85 (0.76–0.91)*	r = 0.49 (0.28–0.65)
	*p* = < 0.0001	*p* *=* *<* *0.0001*	*p* = < 0.0001
9. Options	r = 0.68 (0.51–0.80)	*r* *=* *0.81 (0.69–0.89)*	r = 0.32 (0.07–0.53)
	*p* = < 0.0001	*p* *=* *<* *0.0001*	*p* = 0.014
10. Dochoke	r = 0.65 (0.47–0.78)	*r* *=* *0.81 (0.70–0.89)*	r = 0.41 (0.18–0.60)
	*p* = < 0.0001	*p* *=* *<* *0.0001*	*p* = 0.0009
11. Signs	r = 0.69 (0.54–0.81)	*r* *=* *0.84 (0.73–0.90)*	r = 0.49 (0.27–0.66)
	*p* = < 0.0001	*p* *=* *<* *0.0001*	*p* = < 0.0001
12. Confid	r = 0.39 (0.19–0.56)	r = 0.46 (0.23–0.65)	*r* *=* *0.90 (0.86–0.94)*
	*p* = 0.0003	*p* = 0.0002	*p* *=* *<* *.0001*
13. Explain	r = 0.40 (0.19–0.57)	r = 0.42 (0.17–0.61)	*r* *=* *0.91 (0.87–0.94)*
	*p* = 0.0003	*p* = 0.0012	*p* *=* *<* *0.0001*
14. Clintime	r = 0.35 (0.14–0.53)	r = 0.38 (0.14–0.58)	*r* *=* *0.95 (0.92–0.96)*
	*p* = 0.0015	*p* = 0.0027	*p* *=* *<* *0.0001*
15. Myneeds	r = 0.44 (0.24–0.60)	r = 0.48 (0.25–0.66)	*r* *=* *0.93 (0.90–0.96)*
	*p* = < 0.0001	*p* = 0.0001	*p* *=* *<* *0.0001*
	n = 79	n = 57	*n* *=* *92*
Clinical advice domain (items 1–6)		r = 0.83 (0.73–0.90)	r = 0.42 (0.23–0.59)
		*p* = < 0.0001	*p* = < 0.0001
		n = 59	n = 80
General advice domain (items 7–11)	r = 0.83 (0.73–0.90)		r = 0.49 (0.27–0.67)
	*p* = < 0.0001		*p* = < 0.0001
	n = 59		n = 58
Satisfaction domain (items 12–15)	r = 0.42 (0.23–0.59)	r = 0.49 (0.27–0.67)	
	*p* = < .0001	*p* = < .0001	
	n = 80	n = 58	
Scaling success^a^	5/6 (83.3%)	5/5 (100%)	4/4 (100%)

r ≤ 0.39 correspond to weak correlation; 0.4–0.59 moderate correlation; ≥ 0.6 strong correlation [21]

*CI* confidence interval

^a^Number of correlations that are significantly higher with its own domain, than correlations with other two domains/number of total items in the current domain

In the test–retest reliability estimate, ICC was 0.73 for the General advice domain, 0.82 for the Clinical advice domain and 0.44 for the Patient satisfaction domain.

### Validity

Correlations between S-SWAL-CARE domains, QPP domains and SWAL-QOL Total score are found in Table 5. The Clinical advice domain demonstrated moderate correlations to all QPP domains but one (Physical-technical condition r = 0.21). For the General advice domain moderate correlations were found to all QPP domains (r = 0.35–0.49). The Patient satisfaction domain also demonstrated moderate correlations to all QPP domains. The strongest correlation was found between the Patient satisfaction domain and the Identity-related approach domain (r = 0.64). All S-SWAL-CARE domains demonstrated weak correlations to the SWAL-QOL Total score.

**Table 5:** Spearman correlations between S-SWAL-CARE domains, QPP-domains and SWAL-QOL Total score

S-SWAL-CARE domain	Medical-technical competence (PR)	Physical-technical condition (PR)	Identity-related approach (PR)	Socio-cultural atmosphere (PR)	SWAL-QOL total score
QPP domains					
Clinical advice domain	r = 0.35	r = 0.21	r = 0.32	r = 0.32	r = 0.26
	*p* = 0.001	*p* = 0.160	*p* = 0.003	*p* = 0.004	*p* = 0.023
General advice domain	r = 0.46	r = 0.35	r = 0.44	r = 0.49	r = 0.20
	*p* = < 0.001	*p* = 0.051	*p* = < 0.001	*p* = < 0.001	*p* = 0.130
Patient satisfaction domain	r = 0.49	r = 0.53	r = 0.64	r = 0.52	r = 0.27
	*p* = < 0.001	*p* = < 0.001	*p* = < 0.001	*p* = < 0.001	*p* = 0.010

r < 0.3 weak correlation, 0.3–0.7 moderate correlation, > 0.7 a strong correlation [25]

*PR* perceived reality, dimension in the QPP, *QPP* quality from the Patient’s perspective, *S-SWAL-CARE* Swedish swallowing quality of care questionnaire, *SWAL-QOL* swallowing quality of life questionnaire

Table 6 lists the item-level descriptive statistics as well as the domain statistics for the S-SWAL-CARE. The vast majority of the items showed good variability, where the response ranges spanned mostly all possible values. The total amount of missing responses was 4%. For items 1–11, the percentage of participants who responded “Did not receive any advice” varied between 15 to 40%. For the Clinical advice domain as well as the General advice domain there were no floor or ceiling effects. The Patient satisfaction domain, however, showed high ceiling effects for all items, ranging from 41% (item 13 “Your swallowing clinicians explained everything about your treatment to you”) to 55% (item 15 “Your swallowing clinicians put your needs first”). There were no floor effects for the Patient satisfaction domain.

**Table 6:** Features of score distributions for S-SWAL-CARE, items and domains

S-SWAL-CARE	No. items	No. levels	Range	Median (IQR)	Mean (SD)	Floor n (%)	Ceiling n (%)	Response “Did not receive any advice” n (%)	Missing n (%)
1. Foodeat	NA	6	1–6	3 (3–4)	3.3 (0.9)	1 (1.2)	2 (2.4)	15 (15)	2 (2)
2. Foodavoid	NA	6	1–6	3 (3–4)	3.3 (1.0)	2 (2.6)	2 (2.6)	21 (21)	3 (3)
3. Liqdrink	NA	6	1–6	4 (3–4)	3.7 (1.0)	1 (1.3)	4 (5.2)	19 (19)	4 (4)
4. Liqavoid	NA	6	1–6	3 (2–4)	3.3 (1.1)	1 (1.5)	3 (4.5)	28 (28)	5 (5)
5. Fooddown	NA	6	1–6	3 (3–4)	3.5 (1.2)	1 (1.3)	6 (7.5)	18 (18)	2 (2)
6. Avoidchk	NA	6	1–6	3 (3–4)	3.5 (1.2)	4 (5.1)	6 (7.6)	19 (19)	2 (2)
7. Contact	NA	6	1–6	3 (2–4)	3.0 (1.3)	6 (10.7)	3 (5.4)	40 (40)	4 (4)
8. Goals	NA	6	1–6	3 (3–4)	3.3 (1.2)	4 (5.6)	1 (1.4)	25 (25)	3 (3)
9. Options	NA	6	1–6	3 (2–4)	3.0 (1.2)	6 (9.8)	1 (1.6)	36 (36)	3 (3)
10. Dochoke	NA	6	1–6	3 (2–4)	2.9 (1.4)	9 (14.5)	3 (4.8)	36 (36)	2 (2)
11. Signs	NA	6	1–6	3 (2–4)	3.1 (1.4)	8 (12.9)	4 (6.5)	34 (34)	4 (4)
12. Confid	NA	4	1–4	3 (2–4)	3.0 (1.0)	6 (6.5)	40 (43.0)	NA	7 (7)
13. Explain	NA	4	1–4	3 (2–4)	3.0 (1.1)	12 (13.2)	37 (40.7)	NA	9 (9)
14. Clintime	NA	4	1–4	4 (2–4)	3.2 (1.0)	9 (9.7)	49 (52.7)	NA	7 (7)
15. Myneeds	NA	4	1–4	4 (3–4)	3.3 (1.0)	6 (6.5)	51 (55.4)	NA	8 (8)
Clinical advice domain	6	NA	3–100	47 (40–60)	48.3 (17.7)	1 (1.2)	2 (2.4)	NA	NA
General advice domain	5	NA	0–100	40 (26–53)	41.8 (21.7)	2 (3.3)	1 (1.7)	NA	NA
Patient satisfaction domain	4	NA	0–100	83 (50–100)	70.2 (31.2)	5 (5.3)	28 (29.8)	NA	NA

*IQR* interquartile range, *NA* non-applicable, *SD* standard deviation, S-SWAL-CARE Swedish swallowing quality of care questionnaire

## Discussion

This study describes the adaptation of the SWAL-CARE questionnaire into Swedish. Results revealed high reliability for all domains of the S-SWAL-CARE, with Cronbach’s alpha ≥ 0.90. Even though no CFA could be performed, all items seem to be correctly placed in their respective domains, since all items demonstrated high correlations to their own domain, and lower correlations to the other domains. No Swedish instrument covering the dysphagia population’s perspective of their quality of care has previously been available.

The test–retest analysis demonstrated no statistically significant differences between the first and second assessment, and ICC showed satisfactory correlation for group-level comparison for the Clinical advice and General advice domains, indicating that the results of the S-SWAL-CARE did not change over time. The Patient satisfaction domain showed lower ICC value than the other domains, ICC 0.44 indicating only moderate test–retest reliability. This result was a bit surprising, since the Patient satisfaction domain demonstrated higher ICC in the original study (ICC 0.62) [7]. However, 0.44 and 0.62 are both values corresponding to moderate test–retest reliability. This discrepancy could partly be due to the added response alternative, which might have resulted in a difficulty in discriminating between the response alternatives [16]. The results from the Patient satisfaction domain should therefore be interpreted with some caution due to the somewhat lower ICC, since the results from time to time show a higher variance in this domain.

The analysis regarding the construct validity was performed through correlations between the S-SWAL-CARE and the QPP (convergent validity) and the S-SWAL-CARE and the S-SWAL-QOL (discriminant validity). The results demonstrated a weak correlation between the Clinical advice domain and the Physical-technical condition domain and moderate but still low correlations to the other QPP domains (r = 0.32–0.35), as could be expected. The Clinical advice domain is the domain of the S-SWAL-CARE primarily covering how the patients experience the specific advice regarding which food or liquid consistencies to choose or avoid. Therefore, this should not be strongly correlated to any domains of the QPP, since this more in general covers the quality of care [17, 18]. The low (weak to moderate) correlations between the Clinical advice domain and the QPP domains indicate that the instruments measure two related, but different constructs. The somewhat stronger correlation to the Medical-Technical competence domain indicate that some similarities exist, confirming the a priori hypothesis. The moderate correlations of the General advice domain to the QPP domains (Medical-technical competence, Socio-cultural atmosphere and Identity-related approach domain) also support that the S-SWAL-CARE has a high convergent validity, since this domain covers the more general advice from the swallowing clinician such as advice on “When I should contact a swallowing clinician” and “What to do if I start to choke”. Therefore, the expected results would be higher, yet still moderate correlations compared to the Clinical advice domain. The Patient satisfaction domain also demonstrated moderate, but somewhat stronger correlations (r = 0.49–0.64) to all QPP domains, also as expected, since this domain measures the patient satisfaction and experience of the quality of care in general, showing convergent validity of the instrument. Discriminant validity should indicate that two instruments measure different constructs, thus weak correlations are expected. This was the case in the present study, where weak correlations of all S-SWAL-CARE domains to the SWAL-QOL Total score was found. The results are also similar to the correlations found in the original version, where weak correlations between all SWAL-CARE and SWAL-QOL domains were found (r = 0.05–0.33) [7].

Validation studies often perform Confirmatory Factor Analysis (CFA) in order to investigate the relation between items and domains. However, a CFA demands no missing values in the dataset for the respective study participants. The response alternative “Did not receive any advice”, that was added to the field-testing version of the S-SWAL-CARE, was treated as a missing value. This resulted in complete datasets only for 34 of the study participants, yielding an insufficient number and therefore a proper CFA could not be performed to confirm the factor structure, which could be considered a limitation of the study. However, as all items were strongly correlated to their own domains, with a high scaling success rate, this suggests proper domain structure.

The S-SWAL-CARE was well accepted by the study participants with high compliance, missing-item values were low and few items were found difficult or disturbing, supporting the feasibility of the instrument. For the patients who stated that an item was difficult to understand a majority did not specify why, which can be considered a limitation. For future validation studies it could be valuable to follow up on the reason, in order to better address the potential issues of the item. There were 4 items (When I should contact a swallowing clinician, My treatment options, What to do if I start to choke and Signs that I am not getting enough to eat or drink) where a large proportion of the participants (> 30%) responded “Did not receive any advice”. This is probably due to that most patients receive extensive advice about what food/consistency to eat or not to eat, but the advice does not consistently state what to do when they start to choke, or in which situation they should contact their swallowing clinician. For future studies, it would be of interest to compare the given advice to the patient responses to the S-SWAL-CARE, and to evaluate which items are of most interest for the patients, and perhaps elaborate and add to the advice given to patients in the clinic.

In two of the three domains, the responses covered the full range of scores and floor and ceiling effects were acceptable. However, for the third domain, the Patient satisfaction domain, ceiling effects were found for all items, which can be considered a limitation of the study. Previous research by Jackson et al. [27] and Agoritsas et al. [28] have shown that patients older than 65 and those with better functional status are more likely to be satisfied with the given care. Given that the median age in the patient cohort of the present study is 70 years, this could partly explain the ceiling effect. Considering this aspect, the Patient satisfaction domain might need to be revised. However, top scores in this domain show that the patients are content with the given care, so it might be that this domain is the best measure of quality of care. If a patient is dissatisfied, which would be found in the Patient satisfaction domain, this is a very important aspect to take into consideration. Perhaps these patients might need an extra visit, in order for them to properly understand and be given the opportunity to receive answers to any questions or uncertainties they have regarding the given care.

For validation purposes, a minimum of five respondents per item is required according to Fayers and Machin [16]. Therefore, for the 15-item instrument SWAL-CARE, at least 75 respondents are required. This study included 100 participants in the field testing, which exceeds the minimum number of respondents, and it is therefore deemed sufficient for the validation of this instrument.

The patients in the pilot study requested a response alternative “Did not receive any advice”, for example when they experienced that they had not received advice regarding the specific food or liquid consistency. Patient input is very important in the development of PROMs and PREMs, and the added response alternative could be considered a strength of the study. On the other hand, it could be a limitation to add a response alternative since it may affect the results, given that this leaves room for greater variance of the responses. However, the added response “Did not receive any advice” is not a part of the response scale per se, but accommodates the patient to opt out from answering the item in question. Therefore, the patients who, for example, did not receive advice about “Liquids I should drink” may answer “Did not receive any advice” instead of choosing a response at random, and might therefore result in less variance for this specific instrument. However, the added response alternative resulted in a total of seven response alternatives, and previous literature demonstrate that more than six or seven response alternatives may be difficult for the respondent to discriminate [16].

The S-SWAL-CARE is the first adaptation and validation of the original SWAL-CARE questionnaire. Given the importance of assessing quality of care from the patient’s perspective alongside HRQL, it would be beneficial in clinical care to have accessible tools for evaluating quality of care. Therefore, additional translation and validation studies of the SWAL-CARE should be performed, making the SWAL-CARE questionnaire accessible in more languages than English and Swedish.

### Conclusion

In conclusion, the S-SWAL-CARE can be considered a reliable tool to assess the dysphagia-related quality of care in a mixed dysphagia population. The instrument also demonstrates sufficient convergent and discriminant validity.

## Data Availability

The datasets generated and analyzed during the current study are available from the corresponding author on reasonable request.

## Abbreviations

FEES: Fiberendoscopic evaluation of swallowing
HNC: Head and neck cancer
HRQL: Health related quality of life
ICC: Intra class correlation coefficient
MDADI: M. D. Anderson Dysphagia Inventory
PREM: Patient reported experience measure
PROM: Patient reported outcome measure
QPP: Quality in the patient’s perspective
QOL: Quality of life
SLP: Speech language pathologist
SWAL-CARE: Swallowing quality of care questionnaire
S-SWAL-CARE: Swedish swallowing quality of care questionnaire
SWAL-QOL: Swallowing quality of life questionnaire
